# Change in rheotactic behavior patterns of dinoflagellates in response to different microfluidic environments

**DOI:** 10.1038/s41598-021-90622-8

**Published:** 2021-05-27

**Authors:** Si-Wei Li, Po-Hsu Lin, Tung-Yuan Ho, Chih-hao Hsieh, Chen-li Sun

**Affiliations:** 1 Department of Mechanical Engineering, National Taiwan University, Taipei, 10617 Taiwan; 2 Institute of Oceanography, National Taiwan University, Taipei, 10617 Taiwan; 3 Research Center for Environmental Changes, Academia Sinica, Taipei, 11529 Taiwan; 4 Institute of Ecology and Evolutionary Biology and Department of Life Science, National Taiwan University, Taipei, 10617 Taiwan; 5 Mathematics Division, National Center for Theoretical Sciences, Taipei, 10617 Taiwan

**Keywords:** Motility, Marine biology, Mechanical engineering, Biological physics, Fluid dynamics

## Abstract

Plankton live in dynamic fluid environments. Their ability to change in response to different hydrodynamic cues is critical to their energy allocation and resource uptake. This study used a microfluidic device to evaluate the rheotactic behaviors of a model dinoflagellate species, *Karlodinium veneficum*, in different flow conditions. Although dinoflagellates experienced forced alignment in strong shear (i.e. “trapping”), fluid straining did not play a decisive role in their rheotactic movements. Moderate hydrodynamic magnitude (20 < |**u**_**f**_| < 40 µm s^−1^) was found to induce an orientation heading towards an oncoming current (positive rheotaxis), as dinoflagellates switched to cross-flow swimming when flow speed exceeded 50 µm s^−1^. Near the sidewalls of the main channel, the steric mechanism enabled dinoflagellates to adapt upstream orientation through vertical migration. Under oscillatory flow, however, positive rheotaxis dominated with occasional diversion. The varying flow facilitated upstream exploration with directional controlling, through which dinoflagellates exhibited avoidance of both large-amplitude perturbance and very stagnant zones. In the mixed layer where water is not steady, these rheotactic responses could lead to spatial heterogeneity of dinoflagellates. The outcome of this study helps clarify the interaction between swimming behaviors of dinoflagellates and the hydrodynamic environment they reside in.

## Introduction

Serving as the foundation of the oceanic food chain, plankton play important roles in material cycling and fisheries in the ocean^1^. Contributing to half of the global primary production and regulation of carbon cycling on Earth^1^, phytoplankton form a complex food web with zooplankton, and their trophic interaction is vital to the prosperity of other higher levels of marine life. Studies on understanding how environmental factors such as biochemical and physical forcings control and influence plankton distribution and abundance appear to be substantial in the field^2–6^. For example, the formation of patchiness in planktonic ecosystems was ascribed to non-diffusive processes by some recent research^7^. In particular, the coupling of fluid flow and plankton behavior helped to structure spatial patterns^8^, in which the response timescales of plankton to turbulent advection was determinative^7^. However, this reasoning does not explain the whole picture because plankton heterogeneity could still be found on the order of few centimeters or even sub-millimeter scale where turbulent homogenization of environmental cues was strong^9,10^. To explain the formation of plankton patchiness found in the ocean, several other mechanisms have been proposed: shearing by near-inertial waves, some types of straining, Langmuir circulation, convergent swimming, buoyancy, gyrotactic trapping, localized reproduction, and intrusion of pycnocline^11–13^. As motile microorganisms, plankton can even form aggregation at frontal zones by swimming against vertical currents^14,15^. This seems to suggest that the rheotactic reaction, i.e. the responsive orientation of a microorganism to the flow, can contribute significantly to spatial heterogeneity of planktonic microorganisms. However, our understanding on this directed behavior is still very limited.

In this study, we investigate the rheotactic characteristics of a model dinoflagellate, *Karlodinium veneficum* (CCMP426), by examining their swimming behaviors and orientation dynamics in various flow conditions. Microfluidics have been a useful tool to investigate planktonic biophysics down to a 10 micron level^16–22^. We employed a microfluidic device to provide a manipulative microenvironment so that the trajectory of an individual dinoflagellate can be detected and compared to the local fluid motion. Three different flow conditions were imposed, including: stationary medium, steady flow, and oscillatory flow. The response of plankton to various hydrodynamic cue was then assessed and the rheotactic movement was characterized to elucidate the role of the swimming behavior in promoting accumulation at a specific flow condition. Our results suggest that dinoflagellates *K. veneficum* actively changed their rheotactic movement in response to a highly variable microenvironment, and the unsteadiness of the hydrodynamic condition played a critical role in their swimming behaviors, which could spontaneously contribute to spatial heterogeneity.

## Methods

### Cell culture

The plankton strain used in this study was dinoflagellate *Karlodinium veneficum* (CCMP426), obtained from National Center for Marine Algae and Microbiota. This species was chosen because of the dynamic roles of swimming behavior played in dinoflagellate aggregation^23^ and prey-predator interaction^24,25^. *Karlodinium veneficum* is a mixotrophic dinoflagellate, which can thus be used as prey of copepods or as predator of bacteria to study the influences of hydrodynamic forcing on their motility responses among trophic levels in the future. Batch cultures of the species were grown in 125-ml trace metal clean polycarbonate bottles with 100 ml of F/2-Si medium. All the cultures were kept in a growth incubator with a temperature at 19 °C and illumination at a photon flux density of 100 µmol quanta m^−2^ s^−1^ operated at a 12:12 h light:dark square-wave cycle. The experiments were carried out by using cells grown under their exponential phase and major nutrient concentrations were still replete in the medium. As the cell culture is not axenic from the supplier and the experimental environment was not sterile, bacterial abundance in the medium was likely to be high.

### Configurations of microflow

Dinoflagellates were introduced into a microfluidic device where the fluid flow was either steady or varied periodically with time. The microfluidic device consisted of a 750 µm wide 10 mm long straight main channel connected to a circular microcavity with a radius of 1500 µm. The standard SU8 (SU-8 2150, MicroChem) molding method was employed to make the 460 µm deep microfluidic device from PDMS (Polydimethyl- siloxane).

For the configuration of steady flow, a syringe pump (Fusion200, Chemyx) was employed to deliver the medium into the microfluidic device at a constant volumetric flow rate $\dot{Q}$. The different steady flow condition is described by a dimensionless parameter, the Reynolds number ($Re = 2\dot{Q}/{\upnu }({\text{w}} + {\text{h}})$, where *ν* is the kinematic viscosity of the medium, w = 750 µm and h = 460 µm are the width and height of the main channel, respectively). To impose an oscillatory fluid flow, we used a cam-follower system^26^ that generated a sinusoidal pressure head with an amplitude of 60 mm. Medium containing dinoflagellates was then driven reciprocally in the microfluidic channel. The frequency of the oscillation was fixed at 0.43 Hz, on the same order as gravity waves that contributed to the mixing in the upper ocean^27^. The maximal instantaneous shear rate in the microcavity was on the order of 10 s^−1^, which was one order of magnitude higher than the upper limit of the steady flow used in another study^28^.

### Estimation of flow field

The local flow field in the microfluidic device was estimated separately without dinoflagellates by the µPIV (micro Particle Image Velocity) technique with volume illumination^29–31^. Working fluid was seeded by polyamide microparticles (Dantec Dynamics) with a diameter of 5 µm and a density of 1030 kg m^−3^. The desired quantities of microparticles and working fluid were weighted by a micro balance (MS205-DU, Mettler Toledo) according to a seeding concentration that guaranteed each interrogation cell contained at least 8 particles during the analysis. Small amount of surfactant was then added so that microparticles could wet properly in the aqueous system. The suspension was initially mixed by shaking, and further homogenized by 8–10 min ultrasonic bath (CPX2800, Branson). The acquired image sequence was processed by direct Fourier transform correlation with two-pass grid refining scheme and the deformation of the interrogation samples^29^. Multiplication of the spatial correlation in two 50% overlapping interrogation domains was used to reduce spurious vectors^32^. Table 1 lists the parameters of our µPIV setup.

**Table 1:** Parameters of μPIV setup.

Zone	Objective/adapter	interrogation cell	Seeding concentration	Flow condition
Main channel	5X/1X	16 × 16 px 23.8 × 23.8 μm^2^	7.4 × 10^7^ ml^−1^	all
Cavity	2.5X/1X	16 × 16 px 46.3 × 46.3 μm^2^	1.5 × 10^7^ ml^−1^	stationary, steady flow
Cavity	2.5X/0.63X	10 × 10 px 45.9 × 45.9 μm^2^	7.4 × 10^7^ ml^−1^	oscillatory flow

To reduce error, the steady-flow field was obtained by taking the average among 200 images. For oscillatory flow, the instantaneous full-field flow velocity was determined by taking the phase average of samples from five cycles. The depth over which tracer particles significantly contribute to the measured velocity can be quantified by the depth of correlation (DOC)^33^:

1 $${\text{DOC}} = 2\left\{ {\frac{1 - \sqrt \varepsilon }{{\sqrt \varepsilon }}\left[ {f^{2} d_{p}^{2} + \frac{{5.95\left( {M + 1} \right)^{2} \lambda^{2} f^{4} }}{{M^{2} }}} \right]} \right\}^{1/2}$$

where *ε* is the threshold value that is set to 0.01, *d*_*p*_ is the diameter of tracer particles, *f* and *M* are the f-number and the magnification factor of the objective lens, and λ is the wavelength of incident light. In the current study, the vertical-component of flow velocity was diminutive, if not zero. Although planar flow velocity varied in the vertical direction, the increase in out-of-plane shear could drastically reduce the depth of correlation^34,35^ so that its effect on the µPIV results was intrinsically diminished. For an out-of-plane shear of 6.4 s^−1^ (*Re* = 0.2 in the main channel), the corresponding depth of correlation was 62 µm^36^ and flow velocity only experienced a 7.0% change over the depth of correlation. Under steady and the oscillatory flow, the uncertainties of the estimated flow velocity were ± 0.5% and ± 5.7%, respectively.

**Figure 1:**
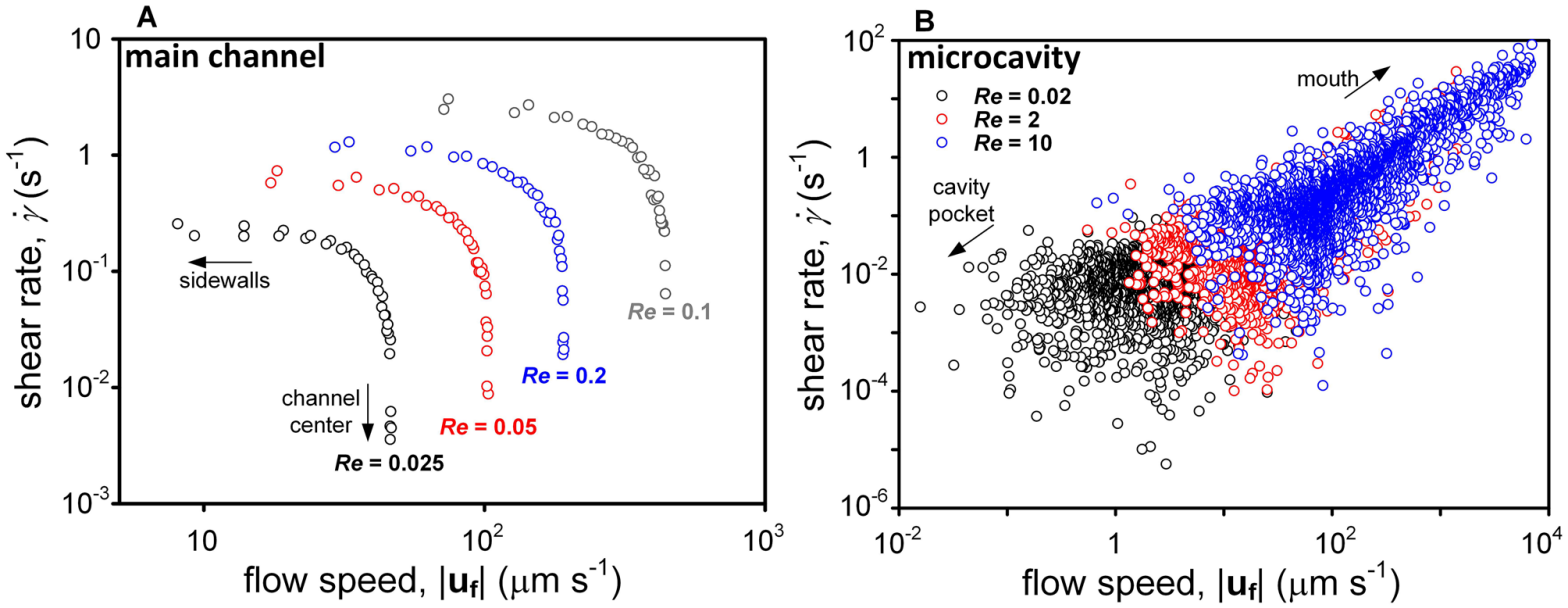
Relationship between flow speed and shear rate in (**A**) the main channel and (**B**) the microcavity under steady flow. In the main channel, high speed appeared in the central region where shear rate was low; low speed was found near the sidewalls where shear rate was high. In contrast, flow speed and shear rate were positively correlated; both increased as moving from the vicinity of pocket bottom toward the mouth.

Our microfluidic device provided two different hydrodynamic microenvironments to dinoflagellates. In the main channel, a high-shear region appeared near the sidewalls where fluid flow was weak, whereas shear rate approached zero at the center where flow was rapid. In the microcavity, on the other hand, flow speed and shear rate were positively correlated (Fig. 1). This difference helped us to discern the influences of flow speed and fluid straining in the swimming behaviors of dinoflagellates. Under oscillatory flow, the instantaneous shear rate in the microcavity approximately varied from 10^−4^ s^−1^ to 10^2^ s^−1^.

### Trajectories and rheotactic behaviors

To track the trajectories of *K. veneficum*, the concentration of dinoflagellates in the microfluidic device was maintained around 10^4^~10^5^ ml^−1^, a concentration that was similar to the level found in harmful algal blooms^37^. Under steady flow, the number of dinoflagellates contained in the cavity and the main channel was 36 ± 5 and 128 ± 11, respectively. Under oscillatory flow, the microcavity had 150 ± 14 dinoflagellates. To ensure the statistical significance of our results, the smallest order of data points used in the analysis was 10^4^.

Image preprocessing began with background subtraction and image binarization with the Otsu’s method^38^ that separates foreground and background by an intensity threshold. The image stack was then analyzed by ImageJ^39^ with the plugin Multitracker to detect the position evolution of individual dinoflagellates. From the given frame rate, the velocity of dinoflagellate **u**_**p**_ was then calculated by the forward differencing scheme. Planktonic velocities that exceed five folds of the average value are regarded as anomalous measurements and rejected. The uncertainty of the absolute planktonic velocity was around 15.6%. For each dinoflagellate, flow velocity **u**_**f**_ of the interrogation window where it was located could be retrieved from the µPIV results. Because the depth of field for planktonic visualization (~57.5 µm) was very close to the depth of correlation for µPIV measurement (~62 µm), the out-of-plane influence was reduced. Therefore, we were able to have a good estimation of the local flow condition that individual dinoflagellate experienced from our two-dimensional PIV diagnosis. In the future, three-dimensional PIV technique^40^ could be employed to obtain information on the full 9 components of fluid shearing and straining. This will open up the possibility for studying more complex interactions, such as how the rheological properties of mucus impact the response of phytoplankton to hydrodynamic forcing.

To evaluate the rheotactic response, the relative swimming velocity (**u**_**pf**_ = **u**_**p**_ − **u**_**f**_) was determined so that the angle between the direction of relative motion of a dinoflagellate and the local flow vector could be calculated from

2 $$\theta = \cos^{ - 1} \left( {\frac{{{\mathbf{u}}_{{{\mathbf{pf}}}} \cdot {\mathbf{u}}_{{\mathbf{f}}} }}{{\left| {{\mathbf{u}}_{{{\mathbf{pf}}}} } \right|\left| {{\mathbf{u}}_{{\mathbf{f}}} } \right|}}} \right)$$

**Figure 2:**
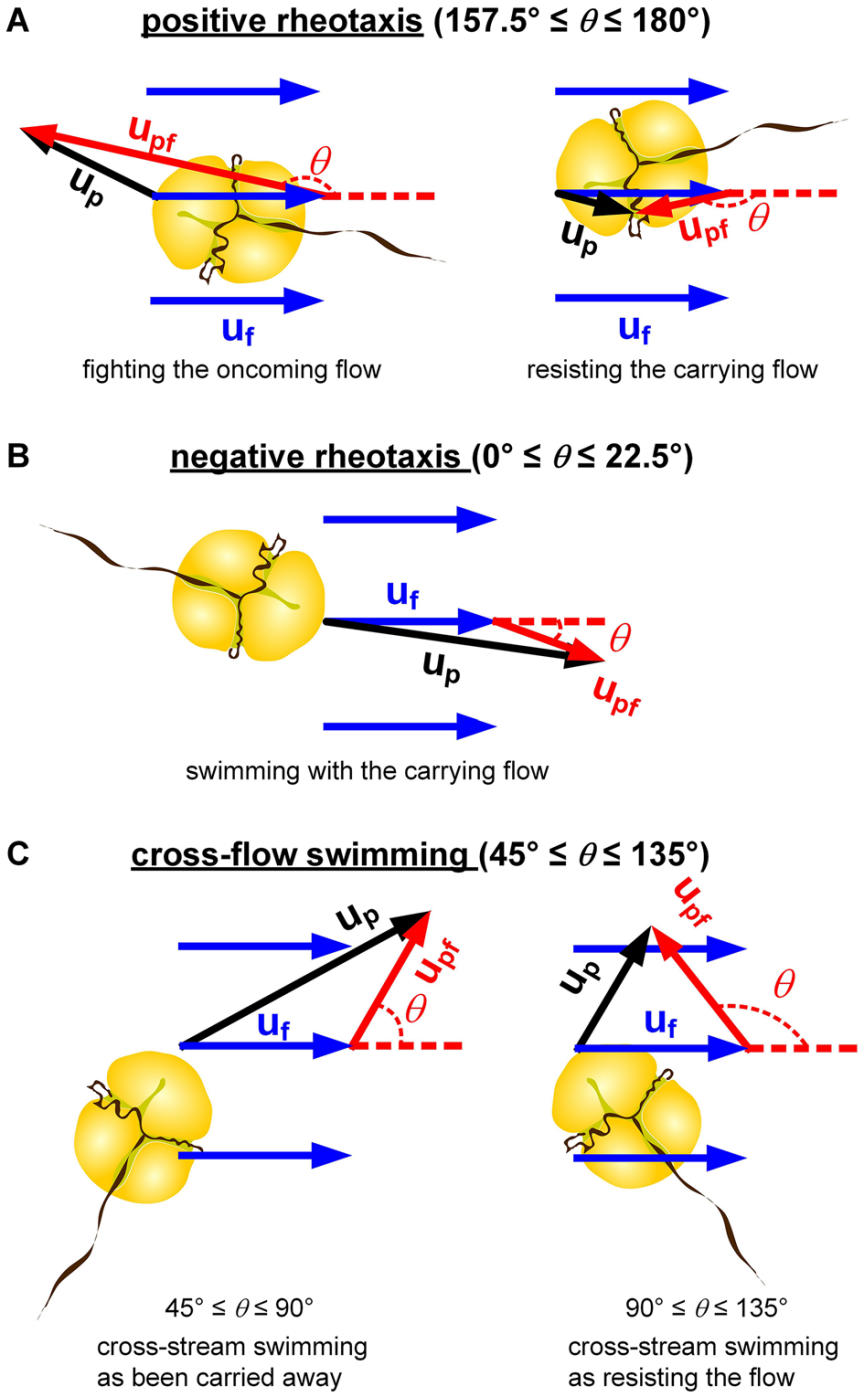
Depiction of rheotactic movement: (**A**) positive rheotaxis^41^ (157.5° ≤ *θ* ≤ 180°) was defined as dinoflagellate moved against fluid flow (left pane) or resisted to move with fluid flow (right pane), (**B**) negative rheotaxis (0° ≤ *θ* ≤ 22.5°) was assigned when dinoflagellate followed fluid flow, (**C**) cross-flow swimming (45° ≤ *θ* ≤ 135°) was referred to as dinoflagellate propelled relatively across the streamlines with downstream (left pane) or upstream orientation (right pane).

The uncertainty of *θ* was around 5°. Due to the difference in temporal resolutions of µPIV and plankton tracking, only data of **u**_**f**_ and **u**_**p**_ taken at 29 given phases of a period were analyzed under oscillatory flow condition. Fig. 2 depicted the ranges of *θ* that were interpreted as positive rheotaxis, negative rheotaxis, and cross-flow swimming.

To smooth out differentiation in the occurrence of flow condition, the conditional probabilities were calculated for various rheotactic responses happening in certain hydrodynamic properties. For example, the conditional probability of positive rheotaxis was the probability of upstream orientations of *K. veneficum* given the occurrence of local flow condition falling within a certain range of |**u**_**f**_| or shear rate (replacing |**u**_**f**_| by $\dot{\gamma }$):

3 $$P\left( {\left. {\theta_{ + } } \right||{\mathbf{u}}_{{\mathbf{f}}} |} \right) = \frac{{P\left( {\theta_{ + } \cap^{{}} |{\mathbf{u}}_{{\mathbf{f}}} |} \right)}}{{P\left( {{\text{all}}^{{}} \theta^{{}} \cap^{{}} |{\mathbf{u}}_{{\mathbf{f}}} |} \right)}}$$

where *θ*_+_ represents a range of angles associated with positive rheotaxis (157.5° ≤  *θ*_+_ ≤ 180°). Similarly, an accumulation index *I*_*A*_ was defined to quantify the deviation of dinoflagellate concentration at a given flow condition:

4 $$I_{A} = \frac{{P\left( {{\text{all}}^{{}} \theta^{{}} \cap |{\mathbf{u}}_{{\mathbf{f}}} |} \right)}}{{P\left( {|{\mathbf{u}}_{{\mathbf{f}}} |} \right)}}$$

In Eq. (4), |**u**_**f**_| represents a certain range of flow speed. If dinoflagellates are evenly disposed, the probability of their appearance in the region within the given range of flow ($P\left( {{\text{all}}^{{}} \theta^{{}} \cap |{\mathbf{u}}_{{\mathbf{f}}} |} \right)$) should be equivalent to the projected-area ratio of this region to the overall evaluation zone ($P\left( {|{\mathbf{u}}_{{\mathbf{f}}} |} \right)$). Therefore, an accumulation index above (below) unity indicated an increase (decrease) in dinoflagellate concentration relative to a homogeneous distribution.

### Swimming in stationary medium

**Figure 3:**
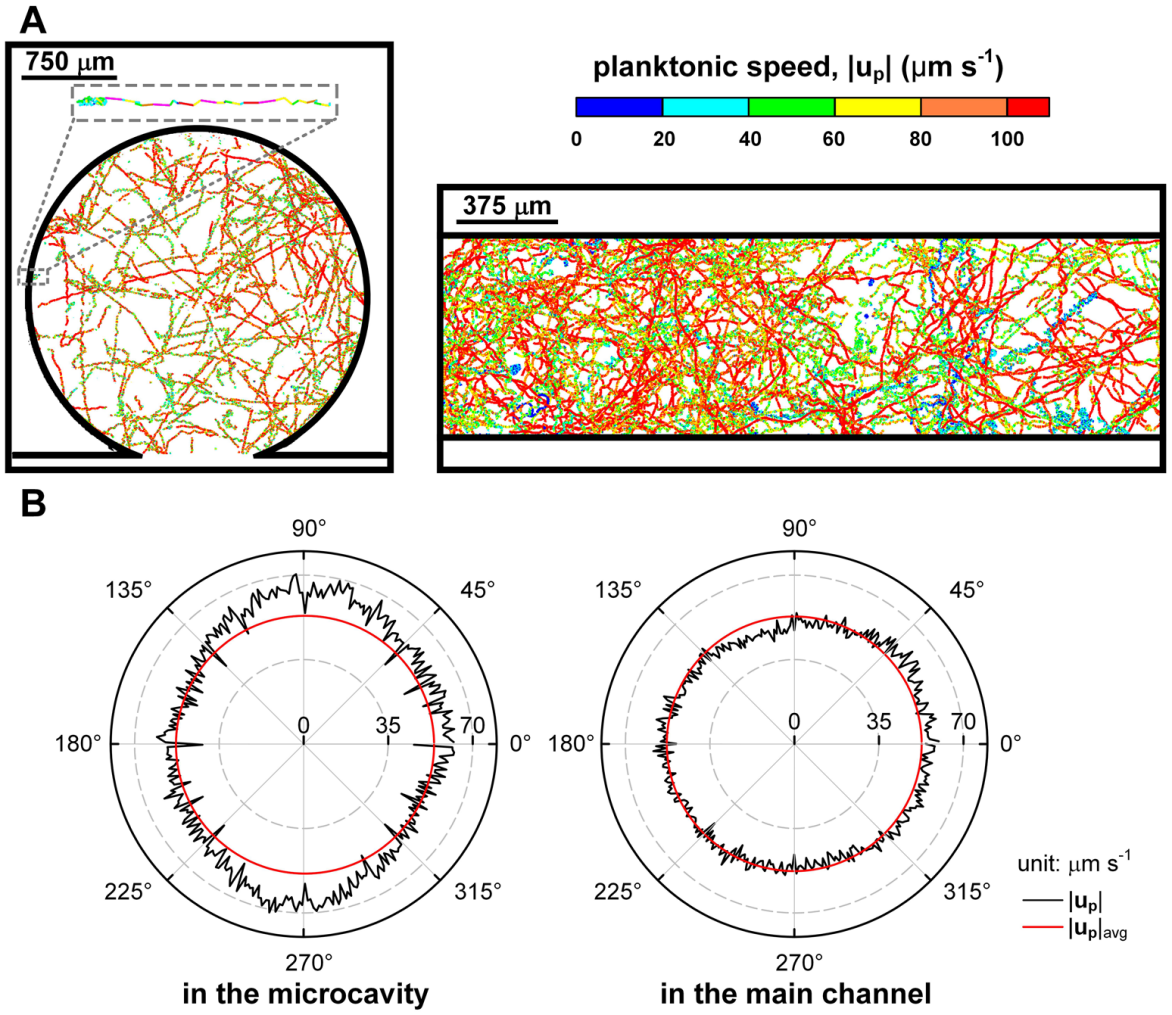
In stationary medium: (**A**) Dinoflagellates confined in the microcavity (dinoflagellate concentration was 6.4 × 10^4^ ml^−1^ and recording duration was 50 s with a frame rate of 100 fps) and the main channel (dinoflagellate concentration was 5.7 × 10^4^ ml^−1^ and recording duration was 100 s with a frame rate of 50 fps) slowed down before changing direction in the near-wall region. The trajectories were colored by the magnitude of their swimming velocity. (**B**) Dinoflagellates showed no preference of orientation, except that they moved slightly faster along the longitudinal direction in the microcavity owing to the microstructure opening. Isotropic swimming was maintained in the main channel.

The trajectories of dinoflagellates *K. veneficum* in stationary medium were first examined to validate that the confinement of the microfluidic device did not alter their swimming behavior^42^. In stationary medium, most *K. veneficum* followed long trajectories and only made sharp turns when they approached obstruction, i.e. the sidewalls (see the insertion of Fig. 3A). Because *K. veneficum* tended to slow down before changing direction, slightly lower swimming speed (cyan or lime sections) was found near the walls in both the microcavity and the main channel (Fig. 3A). The swimming speed along straight trajectory was 58.9 ± 44.9 µm s^−1^, whereas the speed decreased to 31.0 ± 28.9 µm s^−1^ during the turn. On average, the swimming speed of dinoflagellates showed no discrepancy in the microcavity (53.4 ± 38.6 µm s^−1^) and the main channel (52.7±43.5 µm s^−1^). Due to the restriction of our imaging system, the detailed rotating helix of swimming tracks was not resolved^25,43^.

In the microfluidic device, the whole population of dinoflagellates still maintained isotropic swimming, and swimming speed was almost identical in all directions. However, dinoflagellates were found to move slightly faster at a speed of 61.8 µm s^−1^ along the longitudinal direction in the microcavity (Fig. 3B). This value was very close to the average swimming speed of dinoflagellates following straight trajectories. Since the opening of the microcavity faced toward the south, dinoflagellates inside the microcavity were able to travel over a longer distance in the direction around *θ* = 270° without forced diversion by the channel wall. Similarly, dinoflagellates moving from the main channel into the microcavity (about *θ* = 90°) also did not encounter obstacle. If we defined a successful turn by a bend larger than 30°, the average turning rate was 0.29 turn s^−1^ per dinoflagellate in the spans of 80°–100° and 260°–280°, much lower than that (0.83 turn s^−1^) found in other directions. As a result, swimming speed in the longitudinal direction was 15.7% above the mean (Fig. 3B). Besides this small deviation, dinoflagellates in the microfluidic device showed no other change in their swimming characteristics.

## Results

### In steady flow

**Figure 4:**
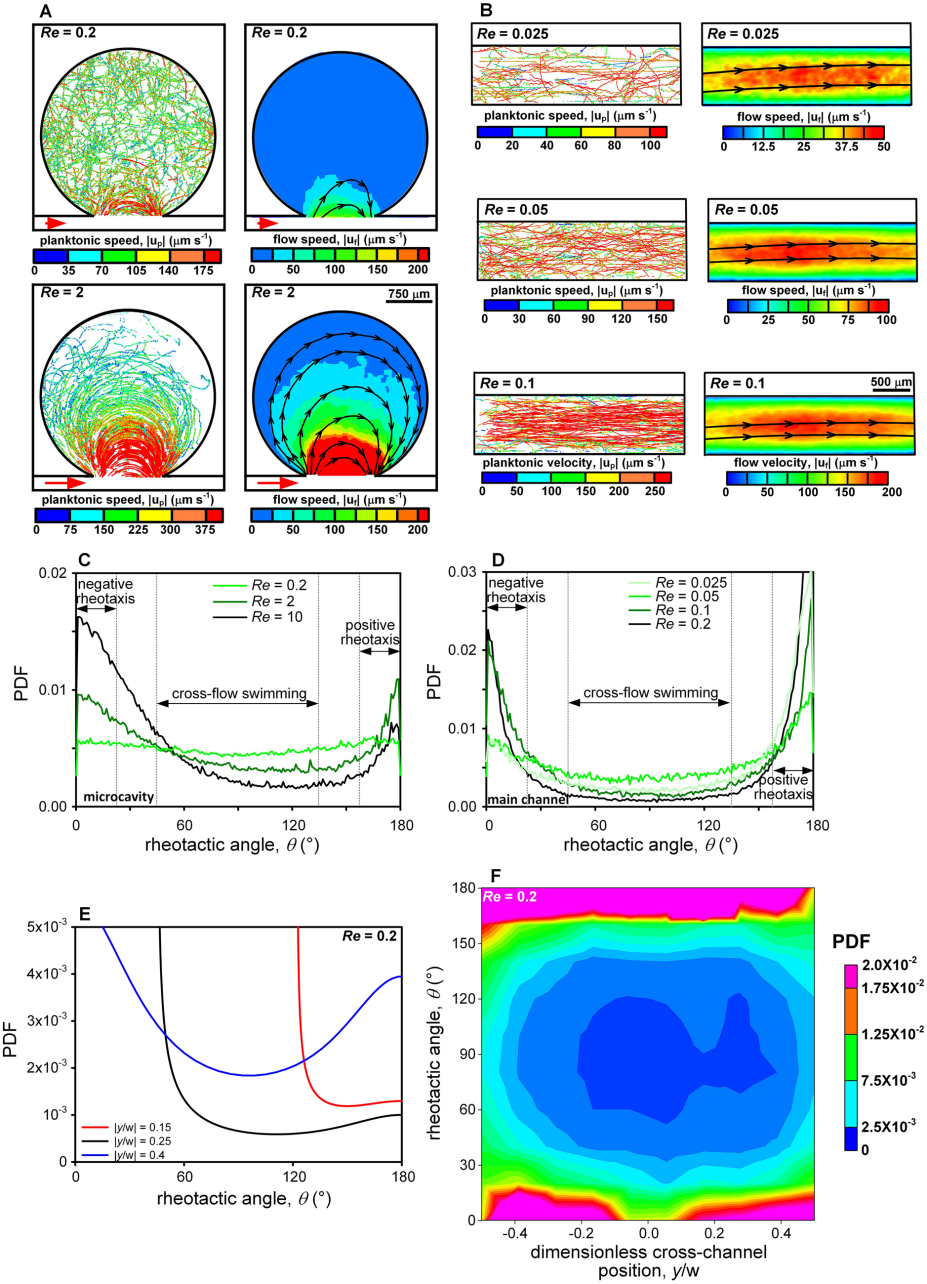
Under the condition of steady flow, dinoflagellate trajectories can be roughly divided into two types: random trajectories in the weak-flow region and streamline trajectories in the strong-flow region. (**A**) In the microcavity, crescent trajectories expanded into the microcavity with the increase of *Re* (*Re* = 0.2: dinoflagellate concentration was 8.4 × 10^4^ ml^−1^ and recording duration was 50 s with a frame rate of 100 fps; *Re* = 2: dinoflagellate concentration was 6.8 × 10^4^ ml^−1^ and recording duration was 25 s with a frame rate of 150 fps). (**B**) In the main channel, straight trajectories were found in the middle of the channel and gradually spread toward the sidewalls as *Re* grew (*Re* = 0.025: dinoflagellate concentration was 6.1 × 10^4^ ml^−1^ and recording duration was 50 s with a frame rate of 50 fps; *Re* = 0.05: dinoflagellate concentration was 4.7 × 10^4^ ml^−1^ and recording duration was 50 s with a frame rate of 50 fps; *Re* = 0.1: dinoflagellate concentration was 5.9 × 10^4^ ml^−1^ and recording duration was 50 s with a frame rate of 100 fps). (**C-D**) The PDFs of the rheotactic angle for dinoflagellates in the microcavity and the main channel both indicated that increase in *Re* resulted in the shifts toward positive and negative rheotaxis. (**E**) Theoretical PDFs of microswimmer orientation with respect to the flow at transverse locations *y*/w = 0.15, 0.25, and 0.4 in the main channel (aspect ratio of microswimmer equal to 1.58)^47^ and (**F**) experimental joint probability distribution of rheotactic angle and position across the main channel at *Re* = 0.2.

Under steady flow, trajectories of plankton could be roughly divided into two types according to local flow speed: streamline trajectories in the strong-flow region and random trajectories in the weak-flow region (Fig. 4). For the Reynolds numbers (*Re*) used in this study, fluid flow entered the microcavity from the left edge of the mouth and left the microcavity at the right edge of the mouth, resulting in circular streamlines. At *Re* = 0.2, strong flow was only found in a small region very close to the cavity mouth, where movement of dinoflagellate was greatly affected by the flow and crescent trajectories were found. As the *Re* increased to 2, the strong-current region expanded much deeper into the microcavity and more dinoflagellates were caught by the large shear to follow crescent paths. This led to a depletion of dinoflagellates in the tranquil zone where fluid flow drastically slowed down due to the wall of the microcavity, and random trajectories, like those appeared in stationary medium, were present. Comparing the dinoflagellate trajectories and the distribution of flow field led to an approximate threshold of flow speed around 75 µm s^−1^ that divided the two types of trajectories, above which the hydrodynamic effect on the movement of dinoflagellates was apparent.

As the Reynolds number increased, *K. veneficum* in the main channel tended to follow streamline trajectories and slightly accumulated in the middle region where flow was relatively high (Fig. 4B). At *Re* = 0.025, most dinoflagellates were able to display autonomous movement. As *Re* increased to 0.1, disorderly paths could only be found in the near-wall region where fluid flow was weak but shear rate was high. At *Re* = 0.1, the maximal shear rate found near the sidewalls was about 1.57 s^−1^, which was comparable to the order of the Kolmogorov-scale shear rate commonly found in the upper mixed layer of the ocean (0.1~1 s^−1^)^8,44^. Therefore, it was reasonable to presume dinoflagellates were able to retain their maneuverability within the eddy field of the surface mixed layer mixed by light to moderate winds. In contrast, most dinoflagellates in the central region were carried away by flow and nearly all trajectories were parallel to the channel walls at *Re* = 0.1. Since flow shear dropped to the minimum at the centerline, shear was apparently not the only hydrodynamic parameter that affected the motility of dinoflagellates^28,45,46^. In the ocean, flow magnitude could also induce changes in the swimming behaviors of *K. veneficum*.

The response of *K. veneficum* to flow was characterized by three different rheotactic movements (definition given in *Method*): positive (upstream) rheotaxis, negative (downstream) rheotaxis and cross-flow swimming. Depending on the flow condition, the swimming behavior of dinoflagellates could switch from one to another. At *Re* = 0.2, PDF of the rheotactic angle was nearly flat in the microcavity (Fig. 4C), revealing that dinoflagellates did not align in any favorable direction and exhibited isotropic swimming in very weak flow. When *Re* increased to 2, two peaks emerged near 0° and 180°, indicating that more dinoflagellates either headed toward the oncoming flow, or simply moved with the stronger current. As *Re* further increased to 10, more dinoflagellates followed the flow and fewer dinoflagellates maintained positive rheotaxis (peak at 0° grew monotonically but the height at 180° diminished). Even cross-flow swimming was preferably done by cutting through the streamlines with downstream orientation at *Re* = 10. In the regime of cross-flow swimming, increasing *Re* resulted in an increase of PDF within 45° < *θ* < 53° but a decrease of PDF for 53° < *θ* < 135° (Fig. 4C).

On the other hand, the hydrodynamic characteristics of the main channel (strong flow with low shear or weak flow with high shear) led to scarce cross-flow swimming^28^, hence the twin-peak PDF distribution of *θ* (Fig. 4D). At *Re* = 0.025, positive and negative rheotaxis were already strong. As *Re* increased to 0.05, PDF associated with cross-flow swimming slightly increased while the peak height around *θ* = 180° reduced. When flow moderately augmented, some dinoflagellates switched from positive rheotaxis to cross-flow swimming. Nevertheless, further increase in *Re* produced a reduction of PDF associated with cross-flow swimming, suggesting that transverse movement became less possible as local flow turned too strong. For *Re* ≥ 0.05, PDF at both *θ* = 0° and 180° grew with the increase of *Re*. Although the enhancement of positive rheotaxis seemed surprising, we found that more dinoflagellates moved vertically with upstream orientation (relative to the planar flow) near the sidewalls as *Re* increased. This vertical motion was detected by out-of-focus dinoflagellates (blurring) in the image sequence and could only be qualitatively described.

The difference between the PDFs of orientation predicted by the Jeffrey orbits^47^ and the joint PDF of dinoflagellate orientation and position across the main channel is apparent (Fig. 4E,F). The deterministic dynamics predicts elongated microswimmers initially located at |*y*/w| = 0.15 and |*y*/w| = 0.25 follow swinging motion and produces a peak orientation around 123° and 46° respectively, and microswimmers close to the sidewalls tumble with slower rotation when aligning downstream (higher peak at 0°) than upstream (lower peak at 180°). These clearly contradict our observation that dinoflagellates exhibited a strong preference of upstream orientation despite their location (Fig. 4F). Near the centerline of the channel, dinoflagellates experiencing large velocity magnitude and low flow shear showed a single orientation peak around 180°. On the other hand, dinoflagellates slightly accumulated near the sidewalls and demonstrate a more diverse orientation. Yet, the leaning of upstream over downstream orientation was evident. Despite the high shear, dinoflagellates located near the mid plane of sidewalls were able to overcome the low out-of-plane shear and move vertically with upstream orientation to retain positive rheotaxis. This preference of vertical movement in intense planar current could help dinoflagellates to escape from being strapped by flow structures, and also possibly expedite the formation of thin patchiness through gyrotactic trapping^46^.

### In oscillatory flow

**Figure 5:**
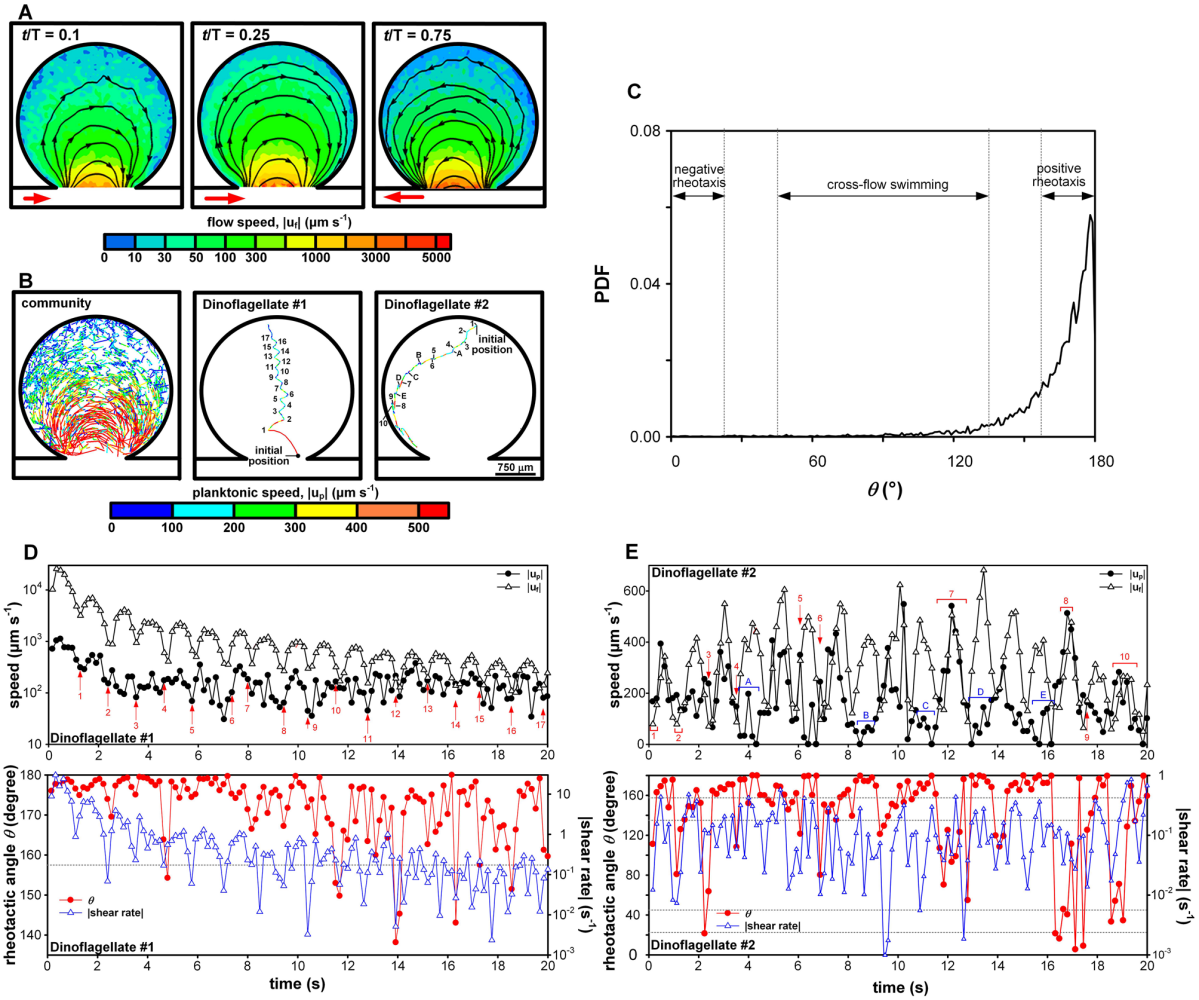
(**A-B**) Zigzag trajectories (dinoflagellate concentration was 6.2 × 10^4^ ml^−1^ and recording duration was 50 s with a frame rate of 100 fps) were found in a crescent zone away from the cavity mouth as the flow field changed with time (a nonlinear color scale was used to adapt to the large span). Nevertheless, individual dinoflagellate did not necessarily follow a winding path. (**C**) PDF of the rheotactic angle showed a very strong tendency of positive rheotaxis for *K. veneficum* in the microcavity under oscillatory flow. (**D-E**) Individual dinoflagellate could regain directional controlling when |**u**_**f**_| and shear rate abated. A large deviation of the moving direction relative to the flow (characterized by smaller *θ*) was usually possible for $\dot{\gamma }$ < 10^–1^ s^−1^ and |**u**_**f**_| < 200 µm s^−1^. The numbers marked in red denote the initiation of turns for Dinoflagellate #1 and the duration of cross-flow swimming for Dinoflagellate #2. The blue letters indicate the halts of Dinoflagellate #2.

In oscillatory flow, *K. veneficum* often followed a zigzag course. While strong flow still controlled their movement near the cavity mouth where maximal flow speed could reach 1000 µm s^−1^ at *t*/T = 0.25 and 0.75 (T = 2.33 s was the period of oscillation), dinoflagellates located deep in the microcavity were found to turn periodically (Fig. 5A,B). To elucidate the feature of locomotion in the time-varying flow, two sampled trajectories of individual dinoflagellate were displayed (Fig. 5B). These two dinoflagellates were chosen because they both moved across regions with wide variety of flow conditions and exhibited some extent of directional controlling; Dinoflagellate #1 passed from high-flow to low-flow regions, and the opposite for Dinoflagellate #2. Dinoflagellate #1 was initially located at the microcavity mouth and swept into the microcavity by the vigorous current. When the flow direction was reversed at *t*/T = 0 or 0.5, the trajectory also had a sharp turn. For *t* < 11 s, the relative orientation of Dinoflagellate #1 only showed small changes with respect to the flow even when local shear rate fell below 1 s^−1^ (#1-#3, #5-#9 in Fig. 5D). In a circular region about 1600 µm from the cavity mouth, the hydrodynamic influence was prominent and Dinoflagellate #1 was forced to bend its course (large *θ* close to 180°). As Dinoflagellate #1 penetrated deeper into the microcavity, the velocity amplitude of intermittent current diminished and Dinoflagellate #1 was able to divert more from the flow direction during a turn and the swing segment also became shorter (#10-#17 in Fig. 5D, small decrease in *θ*). The highest instantaneous flow speed at which Dinoflagellate #1 was able to make a large deflection (i.e. smaller *θ*) was about 600 µm s^−1^, where the local shear rate was only 0.17 s^−1^. The cumulation of these intersecting angles produced a gradual migration over the circular streamlines. Throughout its journey, Dinoflagellate #1 usually moved against the adjacent fluid flow (|**u**_**pf**_|^2^ > |**u**_**f**_|^2^ + |**u**_**p**_|^2^, left pane of Fig. 2A) and exhibited positive rheotaxis.

On the other hand, Dinoflagellate #2 experienced local shear rate lower than 1 s^−1^ throughout its course and demonstrated a mix of swimming behaviors with a strong preference of positive rheotaxis (Fig. 5E). Initially located deep in the pocket where environment was quiescent, Dinoflagellate #2 gradually cut through streamlines by conducting cross-flow swimming periodically (at *t*/T = 0 or 0.5 when flow diminished) for *t* < 4 s (#1-#4 in Fig. 5E). After reaching a location about 2120 µm from the cavity mouth, Dinoflagellate #2 moved along an arc path which almost coincided with a streamline. The sharp turn along this circular segment was limited, as Dinoflagellate #2 shifted between resisting the carrying flow (A-B, D-E in Fig. 5E) and swimming against the current (C in Fig. 5E) or cross-flow swimming (#5-#7 in Fig. 5E). For *K. veneficum*, the resistance to the pushing flow (still positively rheotactic, see right pane of Fig. 2A) was only found in oscillatory flow, since dinoflagellates tended to swim along the carrying current when the flow was steady (negatively rheotactic, see Fig. 2B). This suggests that the rheotactic response of dinoflagellates may pivot on the unsteadiness of the hydrodynamic cue, which made negative rheotaxis unfavorable. Around *t* = 4.16 s, 6.8 s, 8.8 s, 10.88 s, 13.44 s, and 15.84 s (between #5 and #6, A-E in Fig. 5E), |**u**_**p**_| reduced to zero despite of the strong current. Although Dinoflagellate #2 remained in focus in the image sequence, possible maneuverings included vertical movement within the depth of field to penetrate the planar flow or engagement in head-on swimming. The highest instantaneous flow speed in which Dinoflagellate #2 was able to stay at the same location was 498 µm s^−1^ (*t* = 6.4 s in Fig. 5E), a magnitude that was much higher than that found to maintain positive rheotaxis in steady flow. When the flow abated, these “frozen” moments were followed by cross-flow swimming, showing avoidance to surge. For *t* > 16 s, Dinoflagellate #2 was exposed to an intensifying hydrodynamic fluctuation and momentarily carried by the flow (#8, #10 in Fig. 5E).

A PDF peak near 180° confirmed the strong tendency of positive rheotaxis for the whole community of *K. veneficum* in the microcavity under oscillatory flow (Fig. 5C). Despite the temporal variation of flow field, most dinoflagellates spent most of the time either going against the flow, or swimming hard to resist the carrying current. Even when performing cross-flow swimming, dinoflagellates also preferred to cut the streamline by heading toward the oncoming flow (the slight increase in PDF for *θ* > 100°). In oscillatory flow, the PDF level drastically diminished for *θ* < 90° and negative rheotaxis was very rare.

## Discussion

**Figure 6:**
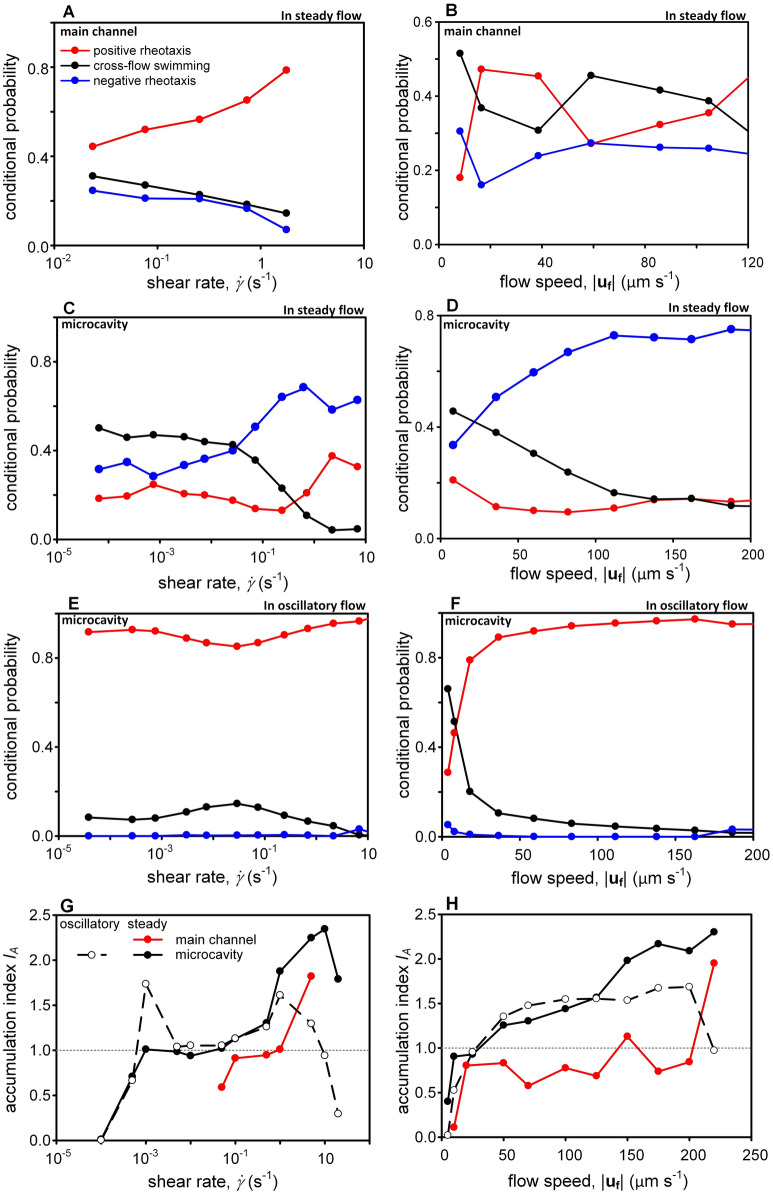
**(A-D)** Under steady flow, rheotactic movement of dinoflagellates depended on both the flow speed and the shear rate. In the main channel where shear rate remained low in the high-flow region, dinoflagellates experiencing a local flow velocity between 20 and 40 µm s^−1^ tended to move against the flow, but were more likely to perform cross-flow swimming when |**u**_**f**_| exceeded 50 µm s^−1^. In the microcavity where local shear rate increased with flow speed, “trapping”^28^ resulted in prominence of negative rheotaxis for $\dot{\gamma }$ > 1 s^−1^. Despite this “weather vane” effect, dinoflagellates in the main channel were able to conduct positive rheotaxis with vertical migration in the near-wall (high shear but weak flow) region. (**E**,**F**) The unsteadiness of the flow completely changed the rheotactic response and positive rheotaxis was always favorable. Cross-flow swimming was possible when |**u**_**f**_| dropped below 10 µm s^−1^. (**G**-**H**) In steady flow, dinoflagellates tended to concentrate in high-flow and high-shear region. In oscillatory flow, dinoflagellates were found to accumulate at instantaneous shear rates of 10^–3^ s^−1^ and 1 s^−1^.

For *K. veneficum*, hydrodynamic cues such as flow speed, shear rate, and unsteadiness could all induce changes in swimming behaviors (Fig. 6). Because of the “weather vane” effect, increasing shear led to a clear decrease in cross-flow swimming under steady flow^28^ (Fig. 6A,C). Two opposite orientations of dinoflagellates were found under this forced alignment, and the differentiation was possibly ascribed to the presence of steric surface interaction and the level of fluid inertia. For $\dot{\gamma }$ ≥ 1 s^−1^, dinoflagellates could be “trapped” and concentrated near the sidewalls of the main channel and the cavity mouth (Fig. 6G). The direct interactions of flagella and solid surfaces could produce a steric mechanism that suppressed long-range hydrodynamic forces^48^. Since dinoflagellates located in the near-wall region of the main channel experienced low fluid inertia, their preferential orientation favored to sustain vertical migration that weakly positive rheotaxis could be maintained with the aid of the steric mechanism (Fig. 6A). Even though dinoflagellates near the microcavity mouth attempted to move vertically, the strong fluid inertia tended to turn their length axis perpendicular to gravity and large drag was exerted by the fluid^49^. Without the steric surface interaction, they were more likely to align with the strong current and positive rheotaxis only experienced a small increase in 0.2 s^−1^ < $\dot{\gamma }$ < 2 s^−1^ (Fig. 6C); the associated flow speed fell between 10 and 1000 µm s^−1^ (Fig. 1). Although dinoflagellates following circular trajectories might experience centripetal force, “shear trapping” remained the dominant mechanism of aggregation. Comparing to the inertia lift, the relative order of flow drag and centripetal forces were 10^4^ and 10^–1^, respectively^50^.

The morphological feature of *K. veneficum* is non-spherical, and their episome and hyposome are divided by cingulum. Hydromechanical signals they generate during swimming depends on both their speed relative to the fluid and their orientation^51,52^, and cross-flow swimming is considered a “noisier” way of locomotion. In the main channel, its dominance in moderate flow (50 µm s^−1^ <|**u**_**f**_|< 110 µm s^−1^, see Fig. 6B) implied that minimizing the hydromechanical disturbance was not prioritized in steady current when shear rate was still low enough to permit turns (corresponding $\dot{\gamma }$ varied between 0.1 and 1 s^−1^, see Fig. 1). Instead, functions such as feeding, exploration, or avoidance could be more important to the locomotion of dinoflagellates. On the other hand, the microcavity provided an interesting analog to an enclosure in a coral reef where water is relatively tranquil. Inside such flow refuge, direction of motion was nearly isotropic and exploration was facilitated by cross-flow swimming for |**u**_**f**_| < 25 µm s^−1^. In the main channel, fluid speed clearly was the key parameter to discern the trends of positive rheotaxis or cross-flow swimming (Fig. 6B). Positive rheotaxis was greatly enhanced for 20 µm s^−1^ ≤|**u**_**f**_| ≤ 40 µm s^−1^, suggesting that moderate hydrodynamic stimulus could trigger upstream exploration. Once flow speed exceeded 50 µm s^−1^, the probability of cross-flow swimming suddenly increased. As flow intensified, dinoflagellates were more likely to move transversely, avoiding direct confrontation to intermediate flow level. When the current was faster than 110 µm s^−1^, “trapping” effect became stronger and movement of dinoflagellates tended to tilt toward the vertical direction and point into the oncoming stream (weakly positive rheotaxis).

Despite the low oscillatory frequency (0.43 Hz), the unsteadiness of hydrodynamic cue resulted in very different rheotactic response of dinoflagellates. Positive rheotaxis overwhelmingly dominated over wide ranges of instantaneous flow speed and shear rate (Fig. 6E,F), as cross-flow swimming often occurred during the flow transition (|**u**_**f**_|< 10 µm s^−1^). Negative rheotaxis was nearly absent, suggesting that dinoflagellates persisted in upstream exploration if local flow environment constantly changed. In addition, the concentration of dinoflagellates peaked at instantaneous shear rates around 10^–3^ s^−1^ and 1–2 s^−1^ (Fig. 6G). Under unsteady flow, “trapping” also became prominent for $\dot{\gamma }$ > 1 s^−1^ and the probability of cross-flow swimming gradually dropped to zero. Nevertheless, the periodic reorientation allowed dinoflagellates to escape from regions with fierce straining motion of the flow. As a result, cell concentration was depleted at extreme shear rates ($\dot{\gamma }$ > 10 s^−1^ or $\dot{\gamma }$ < 10^–3^ s^−1^, see Fig. 6G), revealing that dinoflagellates in oscillatory flow avoided both strong intermittency and very stagnant water. We speculated that the preference to the slightly-disturbed environment could be beneficial to feeding since ambient noise decreased the detection ability of predators^53^, thus leading to the accumulation of dinoflagellates at $\dot{\gamma }$ ~ 10^–3^ s^−1^. As a whole, our results elucidated that the influence of hydrodynamic unsteadiness in planktonic heterogeneity should be treated separately from the steady model of flow commonly employed in numerical simulations^10^.

Either the hydrodynamic cue was steady or unsteady, *K. veneficum* clearly exhibited a tendency to move against the flow. Positive rheotaxis could efficiently increase the relative velocity, therefore expand the exploration range and augment the nutrient uptake by refreshing the fluid circulating around a dinoflagellate^8^. For a cell size around 10–15 µm, the critical velocity above which swimming became effective in enhancing the relative flux was around 15 µm s^−1^. Below this critical velocity, Karp-Boss et al.^54^ argued that dinoflagellates can not maintain a rotational axis parallel to the direction of swimming or direction of shear so that turbulence dominates the advection of nutrient flux. Indeed, positive rheotaxis experienced a sudden jump for 20 µm s^−1^ ≤ |**u**_**f**_| ≤ 40 µm s^−1^ in the main channel (Fig. 6B). However, high shear caused overturning and shifting the axis of the helical motion from the direction of the translational motion, which reduced the flux of nutrient transfer and dinoflagellates were no longer able to increase uptake by swimming^54^. This might provide an ecological reasoning for the depletion of dinoflagellates in $\dot{\gamma }$ > 10 s^−1^ as transitory diversion could be done through cross-flow swimming under oscillatory flow. Because direct quantification of nutrient flux at single-cell level is technically challenging, new approaches should be developed to investigate further whether nutrient uptake is affected by rheotactic response.

For *K. veneficum*, directional controlling was an important feature of their locomotion up to a point that forced alignment took place, providing important functions such as shelter seeking, predator avoidance or range retention. When swimming against a weaker current, dinoflagellates tended to deviate more from the oncoming flow, potentially maintaining a wider range of exploration. However, the speed of fluid flow played a less important role in cross-flow swimming and cutting through the streamlines was always accomplished by a rheotactic angle within ±10° from 90°. This enabled dinoflagellates to escape or search more efficiently in the mode of cross-flow swimming, leading to avoidance of extreme instantaneous shear under oscillatory flow. Our findings might bear important implications of plankton swimming behavior in upper mixed layer of the ocean, where wave action and wind-driven surface current are highly coupled- the combination of positive rheotaxis and cross-flow swimming in such a continuously-changing environment could be beneficial to minimize dispersive losses or increase encounter rate for feeding and mating, which provides adaptive significance. Future studies should take the unsteadiness of the flow environment into account, as dinoflagellates can demonstrate very varied rheotactic behaviors in dynamic water.

## Conclusions

The rheotactic responses of plankton largely depend on the local hydrodynamic environment they interact with, which can ultimately contribute to their accumulation. In steady flow, hydrodynamic stimulus could be an active cue to promote positive rheotaxis or cross-flow swimming for *K. veneficum*, depending on the flow speed. “Trapping” became prominent in strong shear, but interaction between flagella and walls helped dinoflagellates to sustain weakly positive rheotaxis through vertical migration. Without this steric mechanism, majority of dinoflagellates in high straining flow exhibited negative rheotaxis. However, the unsteadiness of flow could mitigate the “trapping” effect, and dinoflagellates were able to divert from its track when the hydrodynamic forcing weakened. The tendency toward positive rheotaxis and cross-flow swimming allowed dinoflagellates to maintain upstream exploration with directional controlling, which could be vital to their survival in open water. In addition, the outcome of this study helped to shed new light on how physical forcing, such as internal waves and tides, could possibly contribute to planktonic aggregation through their rheotactic behaviors. Due to the complex dynamics of the upper mixing layer of the ocean, understanding of the adaptive responses of dinoflagellates to the strength and temporal variation of flow down to the single-cell level could help to clarify the physical mechanisms that structure plankton distribution in a larger scale.
